# Mitochondrial Hyperfusion during Oxidative Stress Is Coupled to a Dysregulation in Calcium Handling within a C2C12 Cell Model

**DOI:** 10.1371/journal.pone.0069165

**Published:** 2013-07-08

**Authors:** Calum J. Redpath, Maroun Bou Khalil, Gregory Drozdzal, Milica Radisic, Heidi M. McBride

**Affiliations:** 1 Cellular Electrophysiology Laboratory, University of Ottawa Heart Institute, University of Ottawa, Ottawa, ON, Canada; 2 Mining Building, Institute of Biomaterials and Biomedical Engineering, University of Toronto, Toronto, ON, Canada; 3 Montreal Neurological Institute, McGill University, Montreal, QC, Canada; University of Medicine and Dentistry of New Jersey, United States of America

## Abstract

Atrial Fibrillation is the most common sustained cardiac arrhythmia worldwide harming millions of people every year. Atrial Fibrillation (AF) abruptly induces rapid conduction between atrial myocytes which is associated with oxidative stress and abnormal calcium handling. Unfortunately this new equilibrium promotes perpetuation of the arrhythmia. Recently, in addition to being the major source of oxidative stress within cells, mitochondria have been observed to fuse, forming mitochondrial networks and attach to intracellular calcium stores in response to cellular stress. We sought to identify a potential role for rapid stimulation, oxidative stress and mitochondrial hyperfusion in acute changes to myocyte calcium handling. In addition we hoped to link altered calcium handling to increased sarcoplasmic reticulum (SR)-mitochondrial contacts, the so-called mitochondrial associated membrane (MAM). We selected the C2C12 murine myotube model as it has previously been successfully used to investigate mitochondrial dynamics and has a myofibrillar system similar to atrial myocytes. We observed that rapid stimulation of C2C12 cells resulted in mitochondrial hyperfusion and increased mitochondrial colocalisation with calcium stores. Inhibition of mitochondrial fission by transfection of mutant DRP1K38E resulted in similar effects on mitochondrial fusion, SR colocalisation and altered calcium handling. Interestingly the effects of ‘forced fusion’ were reversed by co-incubation with the reducing agent N-Acetyl cysteine (NAC). Subsequently we demonstrated that oxidative stress resulted in similar reversible increases in mitochondrial fusion, SR-colocalisation and altered calcium handling. Finally, we believe we have identified that myocyte calcium handling is reliant on baseline levels of reactive oxygen species as co-incubation with NAC both reversed and retarded myocyte response to caffeine induced calcium release and re-uptake. Based on these results we conclude that the coordinate regulation of mitochondrial fusion and MAM contacts may form a point source for stress-induced arrhythmogenesis. We believe that the MAM merits further investigation as a therapeutic target in AF-induced remodelling.

## Introduction

Atrial Fibrillation (AF) is the most common sustained cardiac arrhythmia and worldwide, as populations age, the prevalence of AF will continue to increase[1], [2]. A progressive disorder, AF immediately impairs cardiac performance increasing risks of stroke, dementia, heart failure and death[3], [4]. Current treatment for AF is sub-optimal and will remain so until fundamental mechanisms underpinning AF development and maintenance are better understood[5]. The recent success of catheter ablation in paroxysmal, but not persistent, AF has refocused attention on the importance of atrial tachycardia remodelling (ATR)[5], [6].

Initially paroxysmal, AF provokes functional and structural changes which favour arrhythmia maintenance[6], [7]. The high frequency electrical activity in the fibrillating human atrium *in vivo* is associated *in vitro* with mitochondrial dysfunction, oxidative stress and calcium overload[6], [8]–[11]. If fibrillation persists, surviving atrial myocytes adapt to preserve calcium homoeostasis, a process termed ATR[6], [12]. However, this new equilibrium alters sarcolemmal ion currents and compromises sarcoplasmic reticulum (SR) function promoting triggered activity, re-entry and ultimately perpetuation of AF[6]. Despite the experimental evidence implicating impaired calcium handling in these maladaptive processes, the mechanistic links between mitochondria, oxidative stress and AF-induced remodelling remain largely unexplained[6].

Previous investigations of AF have consistently noted changes in mitochondrial morphology and distribution within atrial myocytes[10], [13], [14]. Confirmation that mitochondria form attachments to SR, the mitochondria associated membrane (MAM), thereby creating calcium microdomains facilitating the rapid uptake and storage of [Ca^2+^]_c_ has implications for ATR which are, as yet, unknown[15], [16]. Mitochondria tether to SR via a protein, mitofusin-2 (Mfn-2), which in addition to regulating mitochondrial fusion and [Ca^2+^]_c_ uptake, maintains mitochondrial respiratory homoeostasis and mediates the SR stress response[17]–[20]. Mitochondria themselves are dynamic organelles responding to short term cellular stress with decreased fission and/or increased fusion, however if stress persists, as occurs during AF, this adaptive response is superceded by mitochondrial fragmentation triggering mitophagy or cellular apoptosis[20], [21].

In addition, mitochondria are the primary intracellular source of reactive oxygen species (ROS), highly reactive small molecules containing unpaired electrons which, under basal conditions, couple excitation contraction and metabolism (ECM coupling) [22]–[24]. However, oxidative stress, wherein excessive production and/or reduced scavenging of ROS occur, causes progressive mitochondrial dysfunction, pro-arrhythmic calcium handling and ultimately apoptosis[22], [25]–[27]. Markers of oxidative stress are elevated in patients who subsequently develop AF[28]. AF results in atrial myocyte stress, increasing mitochondrial ROS formation, ATR and contractile dysfunction[6], [8], [29]–[33]. Anti-oxidants such as the glutathione precursor N-Acetyl Cysteine (NAC) have been demonstrated to be effective, both *in vitro* and *in vivo*, in interrupting this positive feedback cycle[8], [28], [31], [34]–[36].

We hypothesised that the stress of rapid stimulation would increase mitochondrial fusion and promote the formation of MAM in vitro. In addition we postulated that altered mitochondrial plasticity with or without oxidative stress would reversibly alter SR calcium handling and result in increased formation of MAM. In order to test our hypothesis we selected the C2C12 murine myotube model as (i) it has previously been used to investigate mitochondrial plasticity and the acute effects of oxidative stress in vitro, (ii) it is capable of responding to electric field stimulation (EFS) and (iii) it exhibits a myofibrillar system broadly similar to atrial myocytes[37], [38].

## Materials and Methods

### C2C12 mouse myoblast differentiation

**Figure 1:**
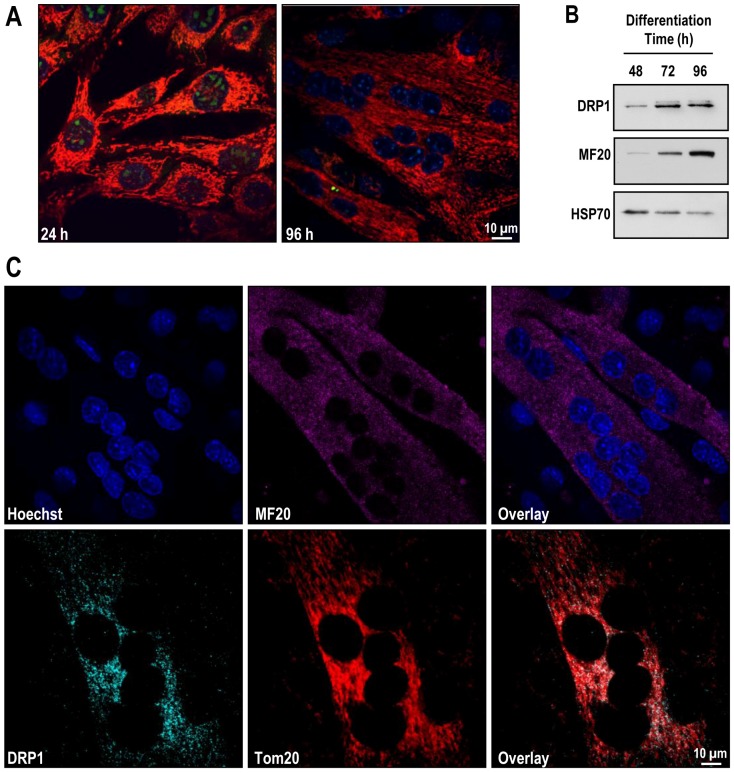
C2C12 myoblasts differentiate into myotubes expressing contractile apparatus and mitochondrial fission machinery. C2C12 myoblasts were cultured and induced to differentiate for 24–96 h at 37°C in an atmosphere of 10% CO_2_. **Panel A:** C2C12 myoblasts differentiate into myotubes. Cells were plated onto coverslips and allowed to reach 70–80% confluence before inducing differentiation. Live images were captured at 405 nm (to visualize DNA, blue), 515 nm (to visualize oxidized hET, green) and 633 nm (to visualize mitochondira, red) on a laser-scanning confocal microscope. **Panel B:** Immunoblots confirming upregulated expression of mitochondrial fission protein DRP1 and contractile protein myosin heavy chain during differentiation in C2C12 myotubes. Cells were harvested, and 50 µg of protein (pooled from 5 separate experiments) were processed for gel electrophoresis. Primary antibodies used were against DRP1, MF-20, and HSP70. **Panel C:** Translocation of DRP1 to the mitochondrial membrane in C2C12 myotubes. Differentiated C2C12 myotubes were fixed, and incubated with primary antibodies against MF20, DRP1 and/or Tom20 and then corresponding Alexa Fluor secondary antibodies. Confocal microscopy images were captured as described before.

Differentiated C2C12 myotubes (Figure 1) were obtained as recently described by Kuo et al[39]. Briefly, C2C12 myoblasts were cultured at 37°C in an atmosphere of 10% CO_2_ in ‘complete’ growth medium (DMEM supplemented with 20% FBS, penicillin-streptomycin and L-glutamine).Once cells reached 70–80% confluence, differentiation was induced by replacing complete medium with low-serum medium (DMEM supplemented with 2% horse serum, penicillin-streptomycin and L-glutamine) and cultured for 24–96 h at 37°C in an atmosphere of 10% CO_2_. Differentiated myotubes, grown onto coverslips (12 mm diameter, 0.16–0.19 mm thickness), were mounted in media with 10 mM Hepes (pH 7.4), 2.5 µg/ml Hoechst, 0.1 µM MitoFluorRed 633, and 5 µM dihydroethidium (hET) 24–96 h post differentiation. Upon oxidation hET (a free radical sensor dye) is cleaved, and the resulting ethidium intercalates with Mitochondrial DNA and, upon high rates of conversion, the dye also fills the cytosol and nucleus as it binds chromosomal DNA. Live images were captured at 405 nm (to visualize DNA, blue), 515nm (to visualize oxidized hET, green) and 633 nm (to visualize MitoFluorRed 633, red) with a 100X NA 1.4 oil immersion objective (Olympus) at 1 airy U on a laser-scanning confocal microscope (IX80; Olympus) operated by FV1000 software version 1.4a (Olympus).

### Protein expression and Immunofluorescence studies

Differentiated C2C12 myotubes were harvested, and 50 µg of protein (pooled from 5 separate experiments) were solubilized in 5x Laemmli sample buffer with 5% β-mercaptoethanol, loaded onto NuPAGE® Novex 4–12% Bis-Tris gradient gels, and separated by gel electrophoresis. For immunoblot analysis, proteins were electrophoretically transferred onto nitrocellulose membranes, followed by blocking with 5% non-fat milk in Phosphare Buffered Saline (PBS)-Tween 20 (137 mM NaCl, 2.7 mM KCl, 100 mM Na_2_HPO_4_, 2 mM KH_2_PO_4_, 0.5% Tween 20, pH 7.4). Primary antibodies used were mouse anti-DRP1 antibody (1∶1000 dilution), mouse anti-myosin heavy chain (MF-20) antibody (1∶5000 dilution), and mouse anti-HSP70 antibody (1∶5000 dilution; used as a loading control). Horseradish peroxidase–conjugated secondary antibodies were used. Signals were developed using Luminata™ Forte Western chemiluminescent HRP substrates.

For immunoflourescence studies, cells were fixed with 4% PFA/PBS for 15 min, quenched with 50 mM ammonium chloride in PBS for 10 min, and permeabilized with 0.1% Triton X-100/PBS for 10 min at room temperature. Cells were then blocked with 5% FBS in PBS for 1 h at room temperature, then incubated (overnight, 4°C) with mouse anti-myosin heavy chain (MF20) antibody (1∶500 dilution), mouse anti-DRP1 antibody (1∶200 dilution) mouse anti-mitofusin 2 (Mfn-2) antibody (1∶200 dilution), rabbit anti-calnexin antibody (5 µg/ml), mouse anti-protein disulphide isomerase (PDI) antibody (1∶100 dilution) and/or rabbit anti-Tom20 antibody (1∶1000 dilution), followed by 3 washes in blocking solution. Cells were next incubated (1∶1000 dilution, 45 min, room temperature) with goat anti-mouse (514 nm) or goat anti-rabbit (647 nm) conjugated Alexa Fluor secondary antibodies, followed by 3 washes with PBS. A final incubation with 2.5 µg/ml Hoechst in PBS (to observe DNA) was performed, followed by 3 washes with PBS before mounting onto slides.

### Electrical Field stimulation (EFS) as a model of “fibrillatory stress”

Monolayers of cultured C2C12 myocytes were placed on gelatin/fibronectin coated coverslips suspended in serum free normal Tyrode solution in a custom designed chamber incubated for 24 hours with 5% CO_2_ at 37°C as previously described[37], [38]. Parallel carbon electrodes connected via platinum wire to a GRASS electrical stimulator continuously applied monophasic square-waves of pulse duration 2 ms, field gradients <8 V/cm without harmful chemical reaction[40]. In order to investigate the effects of “fibrillatory stress”, stimulation protocols include control conditions (0 Hz), simulated physiological conditions (1 Hz) and fibrillatory stress (5 Hz), adapted from Tandon et al[40]. Three days post differentiation, nascent C2C12 myotubes were subjected to stimulation protocols for 24 h at 37°C in an atmosphere of 10% CO_2_. To study SR colocalization, differentiated myotubes were incubated (overnight, 4°C) with mouse anti-PDI antibody (ER marker, 1∶100 dilution) and rabbit anti-Tom20 antibody (mitochondria marker, 1∶1000 dilution), followed by 3 washes with blocking solution. Differentiated myotubes were next incubated (1∶1000 dilution, 45 min, room temperature) with goat anti-mouse (514 nm) or goat anti-rabbit (647 nm) conjugated Alexa Fluor secondary antibodies, followed by 3 washes with PBS. Myotubes were visualized using the confocal microscope as above.

### Studies of mitochondrial plasticity, SR colocalization and calcium handling

Two-days post differentiation, nascent C2C12 myotubes were infected (500 MOI) with dominant-negative dynamin-related protein 1 (DRP1K38E), a mutant form of the mitochondrial fission factor that promotes an elongated mitochondrial reticulum. Empty adenoviral vectors served as control. In order to observe mitochondrial plasticity, differentiated myotubes were loaded with 100 nM tetramethyl-rhodamine ethyl ester (TMRE). Images were captured at 440 nm (to visualize DRP1K38E-CFP expression) and 543 nm (to visualize TMRE, red) using the confocal microscope as above.

To study SR colocalization following DRP1K38E transfection, differentiated myotubes were incubated (overnight, 4°C) with mouse anti-mitofusin 2 (Mfn-2) antibody (1∶200 dilution) and rabbit anti-calnexin antibody (5 µg/ml), followed by incubation (1∶1000 dilution, 45 min, room temperature) with corresponding Alexa Fluor secondary antibodies prior to immunofluorescence studies. Alternatively, intact mitochondria were isolated from differentiated C2C12 myotubes using the mitochondria isolation kit from cultured cells (Pierce), according to manufacturer's instructions. For immunoblot analysis, 30 µg of protein (pooled from 5 separate experiments) were solubilized and separated by gel electrophoresis. Primary antibodies used were against Mfn-2 (3 µg/ml), Tom20 (1∶1000 dilution), SERCA2 (1∶500 dilution), calsequestrin (CSQ, 1∶2500 dilution), calnexin (1 µg/ml), and cytochrome c (Cyt c, 3 µg/ml, loading control). Secondary infrared dye (IRDye800)-labeled antibodies were used for detection on a LI-COR Odyssey infrared imaging system (LI-COR Biosciences). Band intensity was quantified using Odyssey 2.0 software.

To investigate effects of altered mitochondrial plasticity on calcium handling, differentiated myotubes were washed with modified Hanks' buffered saline solution, loaded with 2.5 µM Fluo-4 and 100 nM TMRE, incubated at 37°C in an atmosphere of 10% CO_2_ for 30 min, and then washed with warmed buffer. The methylxanthine caffeine is recognised at millimolar concentration to release Ca^2+^ from intracellular stores during diastole, primarily in a ryanodine receptor (RyR) mediated process involving the SR in skeletal muscle preparations[41], [42]. We used caffeine (4 mM) added to the superfusate to elicit Ca^2+^ waves in C2C12 myotubes during laser scanning confocal imaging to detect release of Ca^2+^ from intracellular stores.

### Studies of oxidative stress and altered redox state

Differentiated C2C12 myotubes were treated with the glutathione synthase inhibitor buthionine sulphoximine (BSO, 200 µM, 24 h) or with the thiol-oxidizing agent diamide (100 µM, 2 h). To assess the effect of oxidative stress on mitochondrial plasticity, differentiated myotubes were loaded with 100 nM TMRE, and images were captured using the confocal microscope as above. In a parallel set of experiments, differentiated myotubes were loaded with 100 nM TMRE and 2.5 µM Fluo-4, and calcium release events were triggered by 4 mM caffeine and detected by laser scanning confocal imaging as above. In order to determine the degree to which oxidative stress was reversible, and to establish if a baseline level of oxidation exists, these protocols were repeated using C2C12 myotubes cultured with the glutathione precursor N-Acetyl Cysteine (NAC 200 µM, 24 h), both pre and post exposure to BSO or DRP1K38E expression.

### Electron Microscopy Studies

C2C12 myoblasts were grown and induced to differentiate on 22 mm glass coverslips. During differentiation nascent C2C12 myotubes were either infected with DRP1K38E (500 MOI) exposed to oxidative stress or EFS-induced “fibrillatory stress”, as previously described, for the final 24 h period prior to processing. Differentiated myotubes were fixed in 2% glutaraldehyde in 0.1M PBS, pH 7.4, post-fixed in 1% osmium tetroxide in PBS, en bloc stained in 3% uranyl acetate, dehydrated in an ascending series of ethanol, and then processed and embedded in Spurr epoxy resin. Thin sections were cut on a Leica UC 6 ultramicrotome, collected on copper grids and counter-stained with lead citrate. Samples were viewed and images taken with a Jeol 1230 TEM equipped with AMT software.

### Statistical Analysis

Results are presented as mean±SE of the mean with the exception of the electron microscopy studies. An unpaired, two-tailed *Student*'*s t-test* was used to evaluate the significance of difference between the calculated means of two groups; multiple comparisons were assessed using one way analysis of variance (ANOVA) via the Prism statistical software package (Graphpad Inc, La Jolla, USA).Some confocal and electron microscopy studies were assessed using one way analysis of variance (ANOVA) or Fisher's exact test where indicated, via Prism as before. A probability value of <0.05 was considered significant.

## Results

### Rapid stimulation results in increased mitochondrial fusion and SR colocalisation

**Figure 2:**
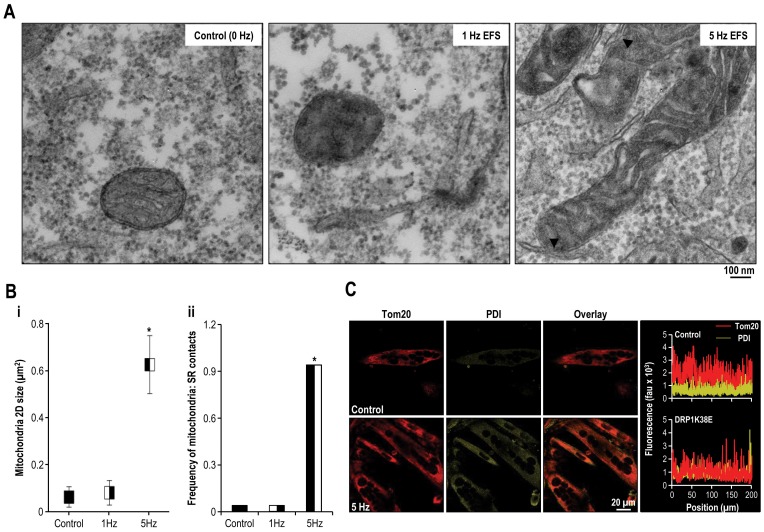
Rapid stimulation promotes mitochondrial fusion, SR colocalisation and increased frequency of SR: mitochondrial contact sites. C2C12 myocytes were induced to differentiate for 72 h, and then were left unstimulated (control) or were subjected to EFS at a frequency of 0 Hz, 1 Hz or 5 Hz for 24 h. **Panel A:** Representative electron micrographs (x25000) of differentiated C2C12 myotubes following 0 Hz (control), 1 Hz or 5 Hz field stimulation. Arrowheads mark mitochondria-SR contact sites. **Panel B:** Objective mean quantitative data for (**i**) mitochondrial 2D area and (**ii**) the occurrence of visible MAM on 2-D electron microscopy. (**i**) Mitochondria 2D size is length multiplied by width. (**ii**) Frequency of mitochondria-SR contact sites is the ratio of the number of mitochondria-SR contacts to the number of mitochondria. **Panel C:** Confocal images of Tom20 and PDI distribution in electric field stimulated C2C12 myotubes. To illustrate colocalisation of mitochondria and ER markers, the corresponding line scans of Tom20 and PDI are drawn on the right. *#* Denotes *p<0.001 Fisher*'*s exact test and one-way ANOVA,* n = 50 all groups.

Exposing differentiated C2C12 cells to sudden and persistent rapid stimulation for 24 h resulted in the formation of elongated mitochondria which colocalised with SR and was associated with an increased frequency of SR:mitochondrial contact sites (Figure 2). There was no significant difference between control cells and those stimulated at 1 Hz in the parameters assessed. However, mitochondrial 2D size increased approximately 5-fold in cells which were stimulated at 5 Hz (Figure 2B) (Mitochondrial 2-D size ( µm^2^): control 0.06±0.04, EFS 1 Hz 0.08±0.05 and EFS 5 Hz 0.63±0.12 *p<0.001* one way ANOVA, n = 50). Similarly, the frequency of SR:mitochondrial contact sites markedly increased subsequent to rapid stimulation (Control and EFS 1 Hz 4%, EFS 5 Hz 94%, *p<0.001* Fisher's exact test, n = 50).

### Altered mitochondrial plasticity, “forced fusion”, increases formation of MAM and results in altered SR calcium handling

**Figure 3:**
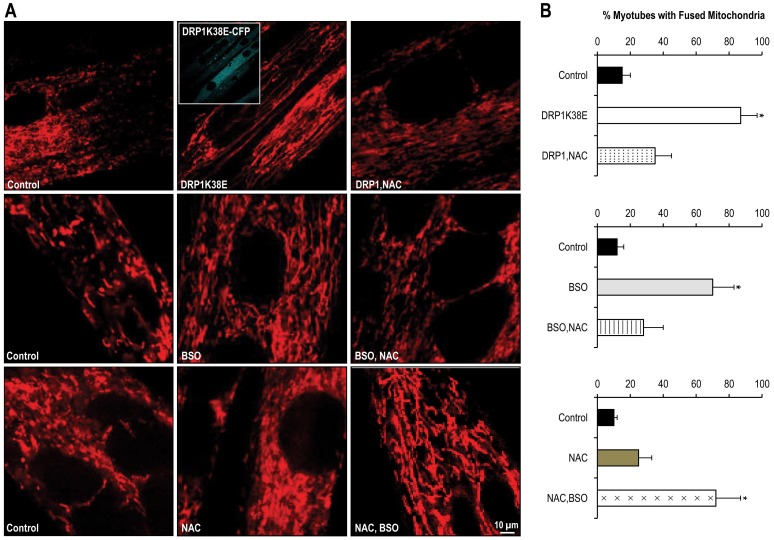
DRP1K38E expression and altered GSH synthesis modulate mitochondrial plasticity in C2C12 myotubes. C2C12 myoblasts were subjected to “forced fusion” or oxidative stress during differentiation. **Panel A:** Confocal microscopy images of C2C12 myotubes expressing DRP1K38E or treated with BSO, NAC or both. **Panel B:** Quantitative Analysis of mitochondria shape is illustrated on the right. 40 images per condition were acquired. Data are mean ±SE. * Denotes *p<0.001 ANOVA,* n = 40 per group.

**Figure 4:**
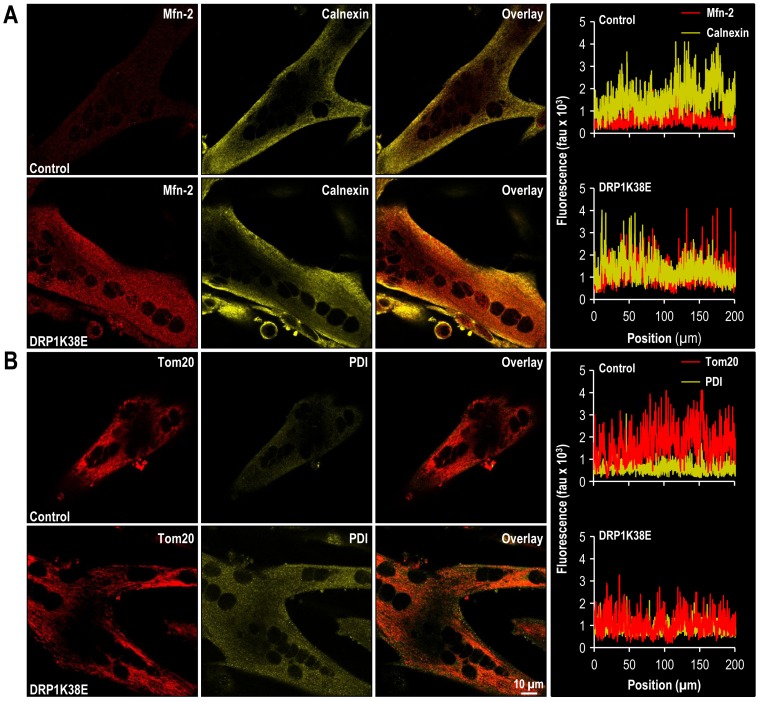
DRP1K38E expression results in a fused mitochondrial reticulum and SR colocalisation in C2C12 myotubes. C2C12 myoblasts were induced to differentiate for 48 h and then were infected with control or DRP1K38E adenoviral vectors for 48 h. **Panel A:** Confocal images of Mfn-2 and calnexin distribution in differentiated C2C12 myotubes. To illustrate colocalization of mitochondria and ER markers, the corresponding line scans of Mfn-2 and calnexin are drawn on the right. **Panel B:** Confocal images of Tom20 and PDI distribution in differentiated C2C12 myotubes. To illustrate colocalization of mitochondria and ER markers, the corresponding line scans of Tom20 and PDI are drawn on the right.

**Figure 5:**
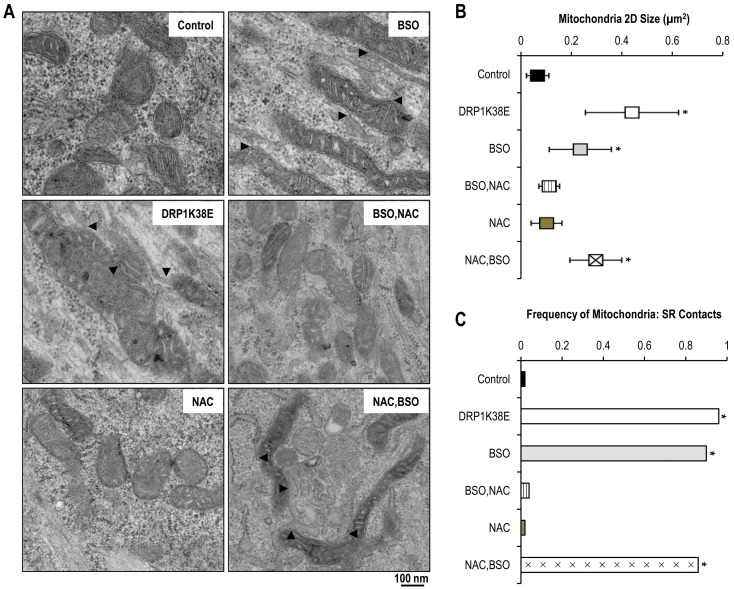
DRP1K38E expression and oxidative stress promote mitochondrial fusion, SR colocalisation and increased SR:mitochondrial contacts. C2C12 myoblasts were induced to differentiate for 48 h and then were infected with DRP1K38E or treated with BSO or NAC. No treatment or empty adenoviral vectors served as control. **Panel A:** Representative electron microscopy images (x15000) of C2C12 myotubes expressing DRP1K38E or having been incubated with BSO, NAC or both. Arrowheads mark mitochondria-ER association sites. **Panel B:** Objective mean quantitative data for mitochondrial 2D area on 2-D electron microscopy. **Panel C:** Objective mean quantitative data for the occurrence of visible MAM on 2-D electron microscopy. * Denotes *p<0.001 ANOVA and Fisher*'*s exact test*, n = 50 all groups.

**Figure 6:**
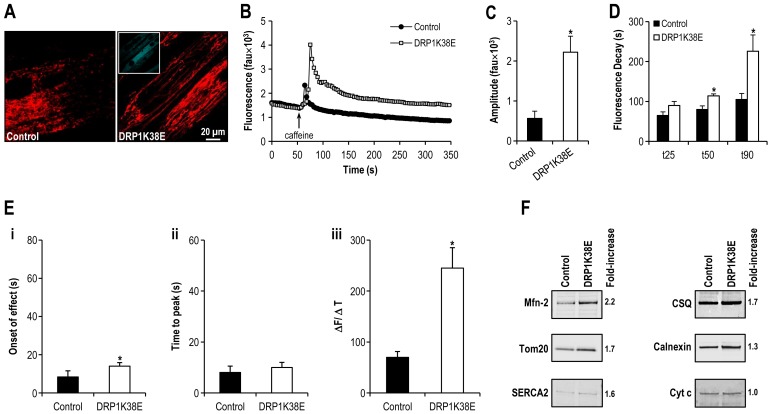
Altered mitochondrial plasticity results in altered SR calcium handling. **Panel A:** Confocal microscopy images of differentiated C2C12 myotubes expressing DRP1K38E, and loaded with TMRE. **Panel B:** Representative tracings of the fluorescence profile of myotubes loaded with fluo-4 prior to the application of caffeine to trigger SR calcium release (DRP1K38E v control). **Panel C:** Objective mean quantitative data for fluorescence resulting from caffeine induced calcium release (DRP1K38E v. control). **Panel D:** Histogram displaying the mean quantitative data relating to the normalization of fluorescence following caffeine application (DRP1K38E v control). **Panel E:** Objective mean quantitative data for fluorescence kinetics profile resulting from caffeine induced calcium release (DRP1K38E v. control). **Panel F:** Immunoblots confirming increased MAM formation in differentiated C2C12 myotubes expressing DRP1K38E. Mitochondria were isolated, and 30 µg of protein (pooled from 5 separate experiments) were processed for gel electrophoresis. Primary antibodies used were against Mfn-2, Tom20, SERCA2, CSQ, calnexin and cytochrome c (cyt c, loading control). * Denotes *p<0.01*–*0.001 ANOVA,* n = 40 per group.

Transfection of differentiated C2C12 myotubes with the dominant negative DRP1K38E was associated with a hyperfused mitochondrial reticulum which colocalised with the SR (Figures 3, 4, 5 and 6A). As compared to control myotubes which had been transfected with empty adenoviral vector, cells expressing DRP1K38E displayed a fivefold increase in fused mitochondria (Figure 3B; control 15±5%, DRP1K38E 87±10% *p<0.001 Student*'*s t-test,* n  = 40 myotubes) and a fourfold increase in 2-D size, as measured on electron micrographs (Figure 5B; mitochondrial 2-D size ( µm^2^): control 0.10±0.04, DRP1K38E 0.43±0.17 *p<0.001 ANOVA,* n = 50).

As compared to control myotubes which had been transfected with empty adenoviral vector, cells expressing DRP1K38E released threefold more calcium upon application of 4 mM of caffeine (Amplitude of caffeine induced calcium release in control myotubes 564±181 fluorescence arbitrary units (fau) v. 2221±397 fau in DRP1K38E myotubes, *p<0.001 Student*'*s t-test,* n = 40 myotubes. Figure 6B, C). There was a significant delay in caffeine response time (Figure 6E i) but not in time to peak caffeine effect (Figure 6E ii) in DRP1K38E myotubes as compared to control (caffeine response time control myotubes 8±3 s v. 14±2 s in DRP1K38E expressing myotubes, *p<0.001*; Time to peak caffeine effect control myotubes 8±2 s v. 10±2 s in DRP1K38E expressing myotubes, *p  = ns*, n = 40 myotubes for all comparisons). However, the first derivative of the caffeine response (ΔF/ΔT, Figure 6E iii) in DRP1K38E myotubes was significantly greater than that observed in control myotubes (caffeine response control myotubes 70±11 fau/s v. 245±40 fau/s in DRP1K38E myotubes, n = 40 myotubes *p<0.001*). In addition to altered calcium release characteristics, subsequent calcium clearance from the cytosol was also significantly prolonged in myotubes expressing DRP1K38E. Early (time to 25% clearance, t_25_), mid (time to 50% clearance, t_50_), and late (time to 90% clearance, t_90_) responses (Figure 6D) were significantly delayed in myotubes with a hyperfused mitochondrial reticulum (Early Ca^2+^ clearance time (t_25_) control myotubes 65±9 s v. 90±10 s in DRP1K38E myotubes; Mid Ca^2+^ clearance time (t_50_) control myotubes 80±9 s v. 114±5 s in DRP1K38E myotubes; Late Ca^2+^ clearance time (t_90_) control myotubes 105±15 s v. 226±41 s in DRP1K38E myotubes; n = 40 *p<0.01* for all comparisons). “Forced fusion”, in the absence of stress, was associated with increased formation of MAM as evidenced by a fused mitochondrial reticulum (Figures 3A, 5B) which colocalised with SR (Figure 4) increased frequency of SR:mitochondrial contact sites (Figure 5C) and increased SR proteins present in mitochondrial pellets isolated from C2C12 cells following transfection with DRP1K38E (Figure 6F).

### Oxidative stress promotes mitochondrial fusion and results in altered SR calcium handling

**Figure 7:**
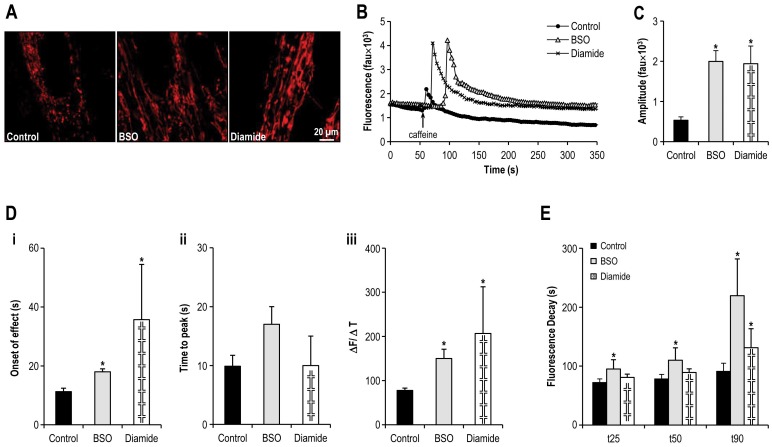
Oxidative stress promotes mitochondrial fusion and results in altered SR calcium handling. **Panel A:** Confocal microscopy images of differentiated C2C12 myotubes having been previously treated with BSO or diamide, and loaded with TMRE. **Panel B:** Representative tracings of the fluorescence profile of myotubes cultured under oxidative stress and loaded with fluo-4 prior to the application of caffeine to trigger SR calcium release (BSO, Diamide v control). **Panel C:** Objective mean quantitative data for fluorescence resulting from caffeine induced calcium release (BSO, diamide v. control). **Panel D:** Objective mean quantitative data for fluorescence kinetics profile resulting from caffeine induced calcium release (BSO, diamide v. control). **Panel E:** Histogram displaying the mean quantitative data relating to the normalization of fluorescence following caffeine application (BSO, diamide v. control). * Denotes *p <0.05*–*0.001 ANOVA,* n = 40 per group.

It has previously been shown that increased cellular levels of oxidized glutathione, which is common during cellular stress, can lead to the activation of the mitochondrial fusion machinery[43]. Consistent with this, inhibition of GSH synthesis with 200 µM of BSO and/or induction of GSH oxidation via diamide (100 µM) altered mitochondrial plasticity promoting a fused reticulum in differentiated C2C12 myotubes (Figures 3, 5 and 7A) [44]–[47]. Oxidative stress was associated with an almost fourfold increase in fluorescence following caffeine application (amplitude of caffeine induced calcium release in control myotubes 534±84 fau v. 1996±269 fau in myotubes incubated with BSO and 1943±438 fau in myotubes treated with diamide, *p<0.001*, ANOVA, n = 40 myotubes Figure 7B,C). Similarly there was a pronounced increase in the caffeine response time (Figure 7D i) but not the time to peak caffeine effect (Figure 7D ii) in myotubes under oxidative stress (caffeine response time control myotubes 11±1 s v. 18±1 s in BSO myotubes (*p<0.001*) and 36±19 s in diamide treated myotubes (*p<0.05*); Time to peak caffeine effect control myotubes 10±2 s v. 17±3 s in BSO myotubes and 10±5 s in diamide treated myotubes (*p = ns*) n = 40 myotubes per group). Again, despite a delayed onset of response to caffeine application as compared with control, there was a significant increase in ΔF/ΔT of the caffeine response (Figure 7D iii) in cells exposed to oxidative stress (caffeine response control myotubes 78±5 fau/s v. 150±21 fau/s in BSO myotubes (*p<0.001*), 207±105 fau/s in diamide treated myotubes (*p<0.05*), n = 40 myotubes per group). Oxidative stress mediated by BSO was consistently associated with altered calcium clearance from the cytosol post caffeine application (t_25_ control myotubes 72±6 s v. 95±16 s in BSO myotubes (*p<0.05*), and 81±6 s in diamide treated myotubes (*p = ns*); t_50_ control myotubes 78±8 s v. 110±21 s in BSO myotubes (*p<0.05*), and 89±6 s in diamide treated myotubes (*p = ns*); t_90_ control myotubes 91±14 s v. 202±62 s in BSO myotubes (*p<0.01*), and 131±32 s in diamide treated myotubes (*p<0.05*); n = 40 myotubes per group. Figure 7E).

### ROS inhibition alters both mitochondrial plasticity and SR calcium handling

The physiological role of low levels of intracellular ROS as signalling molecules is well recognised[22]. In order to compare oxidative stress with reducing stress we devised protocols whereby myotubes were first incubated with oxidizing agents then with reducing agents in addition and vice versa. In this way we aimed not only to determine whether the observed effects of oxidative stress on calcium handling and mitochondrial plasticity could be reversed, but also whether reducing agents themselves had any reversible effects on mitochondrial plasticity and calcium handling.

**Figure 8:**
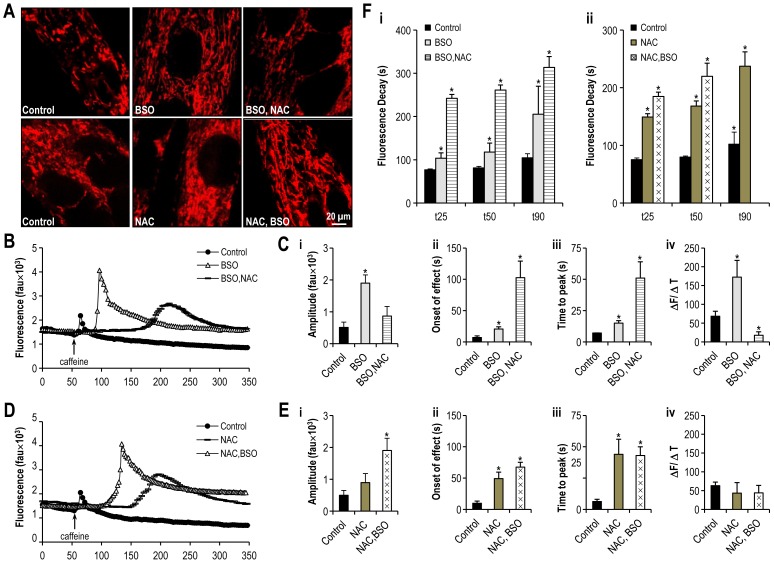
Altering Redox state alters both mitochondrial plasticity and SR calcium handling. **Panel A:** Confocal microscopy images of differentiated C2C12 myotubes having been previously treated with BSO, NAC or both, and loaded with TMRE. **Panels B and D:** Representative tracings of the fluorescence profile of myotubes cultured under oxidative stress or reducing agent or both and loaded with flou-4 prior to the application of caffeine to trigger SR calcium release (BSO, BSO+NAC, NAC, NAC+BSO v. control). **Panels C and E:** Objective mean quantitative data for caffeine induced calcium release and fluorescence kinetics of cells with altered redox states (BSO, BSO+NAC, NAC, NAC+BSO v. control). **Panel F:** Histogram displaying the mean quantitative data relating to the normalization of fluorescence following caffeine application (BSO, BSO+NAC, NAC, NAC+BSO v. control). * Denotes *p<0.001 ANOVA,* n = 20 per group.

Inhibition of GSH synthesis with 200 µM of BSO promoted mitochondrial fusion as before (Figures 3, 5, 7A & 8A). However, as compared to myotubes similarly cultured but with the addition of the glutathione precursor NAC (200 µM) for the final 24 h period of incubation, a less fused reticulum was observed, suggesting reversibility (Figure 8A). In contrast, myotubes cultured solely in the presence of NAC appeared to display a more disconnected reticulum with possibly greater numbers of fragmented mitochondria, suggestive of reduced fusion or increased fission or both. Subsequent to the addition of BSO to culture media for 24 h the mitochondria appeared to return to a more fused network (Figure 8A).

Inhibition of glutathione synthase by BSO was again associated with an almost fourfold increase in fluorescence which was reversed by the addition of NAC (amplitude of caffeine induced calcium release in control myotubes 513±164 fau v. 1898±258 fau in myotubes incubated with BSO (*p<0.001*) and 865±300 fau in myotubes treated with BSO+ NAC (*p = ns*), ANOVA, n = 40 myotubes Figure 8B, C). In contrast, the addition of NAC to myotube media resulted in an approximate three to fivefold potentiation of the delay in caffeine response (Figure 8C ii) and the time to peak (Figure 8C iii) caffeine response (caffeine response time control myotubes 7±3 s v. 21±4 s in BSO myotubes (*p<0.001*) and to 103±26 s in BSO + NAC treated myotubes (*p<0.001*; time to peak caffeine effect control myotubes 7±0 s v. 15±2 s in BSO myotubes (*p<0.001*) and 51±13 s in BSO+ NAC treated myotubes (*p<0.001*), n = 40 myotubes per group). The BSO-mediated increase in ΔF/ΔT of the caffeine response was reversed in those myotubes in which NAC was added to the media prior to experimentation (caffeine response control myotubes 68±13 fau/s v. 172±45 fau/s in BSO myotubes (*p<0.001*), as compared to 18±9 fau/s in BSO+ NAC treated myotubes (*p<0.001*), n = 40 myotubes per group. Figure 8C iv). In keeping with the marked delay in fluorescence following reversal of oxidative stress, recovery from fluorescence was similarly delayed (Figure 8F i), particularly in the early to mid phase of recovery (t_25_ control myotubes 77±2 s v. 104±12 s in BSO myotubes (*p<0.001*), and 242±9 s in BSO+ NAC treated myotubes (*p<0.001*); t_50_ control myotubes 81±3 s v. 118±21 s in BSO myotubes (*p<0.001*), and 261±12 s in BSO+ NAC treated myotubes (*p<0.001*); t_90_ control myotubes 105±9 s v. 206±64 s in BSO myotubes (*p<0.001*), and 313±25 s in BSO+ NAC treated myotubes (*p<0.001*), n = 40 myotubes per group).

Addition of the glutathione precursor NAC to myotubes media during culture was associated with an approximate 80% increase in fluorescence which was further doubled by the addition of BSO to culture media (amplitude of caffeine induced calcium release in control myotubes 498±152 fau v. 894±285 fau in myotubes incubated with NAC (*p<0.01*) and 1902±387 fau in myotubes treated with NAC+ BSO (*p<0.001*), ANOVA, n = 60 myotubes, Figure 8E i). Again, the addition of NAC to media resulted in an approximate fivefold potentiation of the delay in caffeine response (Figure 8E ii) and a sevenfold increase in the time to peak (Figure 8E iii) caffeine response (caffeine response time control myotubes 10±3 seconds (s) v. 49±10 s in NAC myotubes (*p<0.001*) and to 67±8 s in NAC+ BSO treated myotubes (*p<0.001*); Time to peak caffeine effect control myotubes 6±2 s v. 44±12 s in NAC myotubes (*p<0.001*) and 43±7 s in NAC+ BSO treated myotubes (*p<0.001*), n = 40 myotubes per group). There was no significant effect of NAC on ΔF/ΔT of the caffeine response (Figure 8E iv) and the expected BSO mediated increase in ΔF/ΔT was not observed in the NAC pre-treated myotubes (caffeine response control myotubes 63±10 fau/s v. 43±28 fau/s in NAC myotubes and 44±20 fau/s in NAC+ BSO myotubes (*p = ns* for all comparisons), n = 40 myotubes per group). Addition of NAC to culture media resulted in a significant delay to clearance of calcium from the cytosol (Figure 8F ii), and it was not possible to observe complete recovery in myotubes exposed to NAC+ BSO (t_25_ control myotubes 75±3 s v. 149±6 s in NAC myotubes (*p<0.001*), and 185±7 s in NAC+ BSO treated myotubes (*p<0.001*); t_50_ control myotubes 80±2 s v. 168±9 s in NAC myotubes (*p<0.001*), and 220±23 s in NAC+ BSO treated myotubes (*p<0.001*); t_90_ control myotubes 102±21 s v. 237±25 s in NAC myotubes (*p<0.001*), n = 40 myotubes per group).

### Altering mitochondrial plasticity, fibrillatory and oxidative stress similarly result in enlarged mitochondria with increased frequency of SR: mitochondrial contacts

**Figure 9:**
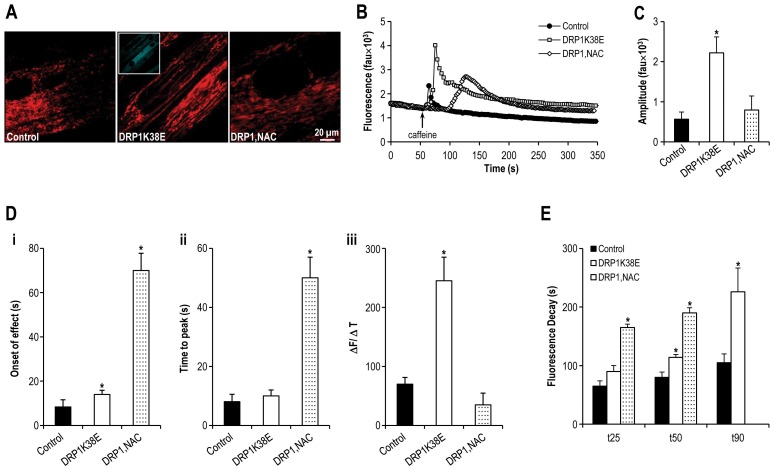
The reducing agent NAC reverses the alterations in SR calcium release induced by ‘forced fusion’. **Panel A:** Confocal microscopy images of differentiated C2C12 myotubes expressing DRP1K38E or treated with NAC, and loaded with TMRE. **Panel B:** Representative tracings of the fluorescence profile of myotubes loaded with fluo-4 prior to the application of caffeine to trigger SR calcium release (DRP1K38E, DRP1K38E+NAC v control). **Panel C:** Objective mean quantitative data for fluorescence resulting from caffeine induced calcium release (DRP1K38E, DRP1K38E+NAC v. control). **Panel D:** Objective mean quantitative data for fluorescence kinetics profile resulting from caffeine induced calcium release (DRP1K38E, DRP1K38E+NAC v. control). **Panel E:** Histogram displaying the mean quantitative data relating to the normalization of fluorescence following caffeine application (DRP1K38E, DRP1K38E+NAC v. control). * Denotes *p<0.001 ANOVA,* n = 40 per group.

Transfection of dominant-negative mutant DRP1K38E resulted in a more fused mitochondrial reticulum composed of fewer, larger mitochondria as compared to control (Figures 3, 4, 5, 6 and 9). Inhibiting mitochondrial fission in this way resulted in a greater than fourfold increase in mitochondrial 2-D cross-sectional area with a significant increase in observed SR: mitochondrial contact points (Figure 5). Similarly, oxidative stress resulted in a significant enlargement of mitochondria, associated with a more fused reticulum (Figures 3, 7, 8, 9) and promoted formation of a greater number of visible SR: mitochondrial contacts (Figure 5C). Interestingly, the addition of the reducing agent NAC had no effect on mitochondrial fusion under control conditions but reversed not only the fusion induced by BSO but also infection with DRPK138E (Figures 3, 5, 8, and 9). In addition, NAC reversed the effect of ‘forced fusion’ on caffeine induced calcium release (Figure 9).

Addition of the glutathione precursor NAC to myotubes previously infected with DRP1K38E was associated with a reduction in fluorescence to control levels (amplitude of caffeine induced calcium release in control myotubes 564±181 fau v. 2221±397 fau in myotubes incubated with DRP1K38E (*p<0.001*) and 795±350 fau in myotubes treated with DRP1K38E+ NAC (*p = ns*), ANOVA n = 40 myotubes Figure 9B, C). Again, the addition of NAC to media resulted in an approximate fourfold potentiation of both the delay in caffeine response (Figure 9D i) and in the time to peak (Figure 9D ii) caffeine response (caffeine response time control myotubes 8±3 s v. 14±2 s in DRP1K38E myotubes (*p = ns*) and to 70±8 s in DRP1K38E+ NAC treated myotubes (*p<0.001*); Time to peak caffeine effect control myotubes 8±2 s v. 10±2 s in DRP1K38E myotubes (*p = ns*) and 50±7 s in DRP1K38E+ NAC treated myotubes (*p<0.001*), n = 40 myotubes per group). The addition of NAC significantly reversed the effect of DRP1K38E on ΔF/ΔT (Figure 9D iii) of the caffeine response (caffeine response control myotubes 70±11 fau/s v. 245±40 fau/s in DRP1K38E myotubes and 35±20 fau/s in DRP1K38E+ NAC myotubes (*p<0.001* for all comparisons), n = 40 myotubes per group). Addition of NAC to culture media resulted in a significant delay to clearance of calcium from the cytosol (Figure 9E). Indeed, it was not possible to observe complete recovery in myotubes exposed to DRP1K38E+ NAC (t_25_ control myotubes 77±2 s v. 104±12 s in DRP1K38E myotubes (*p = ns*), and 242±9 s in DRP1K38E+ NAC treated myotubes (*p<0.001*); t_50_ control myotubes 81±3 s v. 118±21 s in DRP1K38E myotubes (*p<0.01*), and 261±12 s in DRP1K38E+ NAC treated myotubes (*p<0.001*); t_90_ control myotubes 105±15 s v. 206±64 s in DRP1K38E myotubes (*p<0.001*), n = 40 myotubes per group).

## Discussion

With these studies we provide further evidence of the intimate relationship between mitochondrial fusion, oxidative stress, and altered SR calcium handling in murine myocytes. Increased MAM formation, via inhibition of mitochondrial fission or triggered by oxidative stress, was associated with increased caffeine induced calcium release with preservation of release kinetics and delayed cytosolic clearance of calcium. Further corroboration of the redox sensitive nature of intracellular calcium transporters is provided by the slowing of SR calcium release and the potentiation of delayed calcium clearance in the presence of the glutathione precursor NAC.

Mitochondria are increasingly recognised to perform a number of vital functions in mammalian cells. In addition to their long appreciated role in cellular respiration, the discovery of MAM has linked mitochondrial plasticity, ECM coupling and cellular stress responses. The central role of rapid bidirectional calcium signalling between SR and mitochondria at the MAM offers tremendous opportunities to improve our understanding of various cardiomyocyte pathophysiologies. We sought to investigate the relationship between fibrillatory and oxidative stress, mitochondrial fusion and calcium handling with simple experiments in order to identify a possible link between AF-induced cellular stress and subsequent atrial tachycardia remodelling.

As mitochondria cannot be generated de novo, they are perpetually being repaired and recycled in a dynamic equilibrium between opposing processes of fission and fusion[20]. This plasticity is required in order to preserve both mitochondrial and myocyte integrity[18]. Fusion produces elongated interconnected mitochondria and formation of mitochondrial networks facilitates the transmission of calcium signals and membrane potential across individual cells[20]. Mitochondrial fission generates numerous morphologically and functionally distinct isolated mitochondria and can be physiological, as a prelude to mitophagy, or pathological in response to rapid increases in [Ca^2+^][20], [48]. Dysfunctional mitochondrial plasticity results in increased sensitivity to apoptotic stimuli and is pro-arrhythmogenic, however the importance of mitochondrial plasticity in human heart disease is only just being recognised[18], [49], [50].

The Mitochondria-Associated Membrane is the physical association of juxtaposed SR and mitochondrial membranes which facilitates privileged inter-organelle communication performing several physiological functions[15], [51]–[53]. Both inter-organelle proximity and formation of MAM tethers are dynamic, remodelling in response to local [Ca^2+^]_c_ [53]–[55]. The first direct MAM tether identified in the mammalian system was Mitofusin-2 (Mfn-2), a large trans-membrane protein residing in the outer mitochondrial membrane (OMM) pivotal to mitochondrial fusion[17]. As SR structural integrity and bidirectional calcium signalling at the MAM are Mfn-2 dependent, the MAM may also perform critical roles in calcium buffering and the regulation of cellular respiration during stress[17], [56]–[58].

Mitochondria generate ATP by oxidative phosphorylation with calcium and ROS acting as signalling molecules in a bioenergetic homoeostasis involving mitochondria, SR and the nucleus[22], [24]. Mitochondrial respiration is also the primary source of ROS within myocytes which, under normal conditions, remain counterbalanced by calcium-dependent production of reducing agents[22], [59]. Accumulation of ROS impairs myofibrillar calcium handling and has been implicated in contractile dysfunction and AF-remodelling[30], [36]. Altered phosphorylation of sarcolemmal ion currents modulates whole cell calcium entry and extrusion, while oxidation of the SR calcium transporters RyR and SERCA modify SR calcium release and re-uptake[26], [27], [31]. Experimentally induced mitochondrial ROS production induces pro-arrhythmic SR calcium release[60]. If metabolic stress is not relieved, oxidative stress uncouples mitochondria, disrupts cytosolic proteins and nuclear DNA resulting in cellular dysfunction and ultimately apoptosis[22], [25], [26], [61].

Confirmation of the existence of microdomains of Ca^2+^ and ROS at the MAM suggest the possibility of crosstalk between respiration, oxidative stress, mitochondrial plasticity and calcium handling[15], [53], [60], [62], [63]. Recent descriptions of stress induced mitochondrial hyperfusion (SIMH) provide further credence to the possibility of such crosstalk[21]. Mitochondria initially respond to a variety of cellular stresses by forming a fused network in an apparent protective effect[20]. Mitochondrial function is maintained temporarily under adverse circumstances; however if stress persists mitochondrial fragmentation and ultimately cell death occur[20], [21]. In related work, ROS induced disulphide switching of Mfn-2 promoted mitochondrial fusion in a GTP dependent process directly linking oxidative stress and mitochondrial fusion[43]. Recent data suggests that stress induced phosphorylation of drp1 by PKA inactivates fission, promoting formation of a fused mitochondrial network[20], [64]. This hyperfused reticulum appears resistant to mitophagy, conferring temporary protection to cells from death triggers, ‘buying time’ for other homoeostatic mechanisms to alleviate stress and thus preserve cellular integrity[20]. It is not known which combination(s) of factors promote switching from SIMH to fragmentation nor the circumstances which determine the threshold for death triggers, although one should not be surprised to learn that emerging data supports a regulatory role for Mfn-2 mediated bidirectional calcium signalling in SR stress responses[17], [19].

Our results support a role for hyperfusion in response to stress and broadly speaking appear in agreement with those of the Dorn laboratory[18], [65]. Similarly, altered calcium handling by the SR and/or mitochondria associated with increased MAM formation, either as a result of oxidative stress or unopposed fusion, suggests that Mfn-2 is critical to crosstalk. Caffeine induced SR calcium release into mitochondria has previously been reported and is related to proximity of the MAM and the rate of SR emptying[15], [66], [67]. Opposing sides of the MAM preferentially locate with anchoring of outer and inner mitochondrial membrane permitting privileged access to the respiratory complexes[68]. Prevention of mitochondrial calcium overload has previously been shown to rescue *in vitro* models of pathological tachycardia and cardiomyopathy[69]–[72]. Mitochondrial calcium overload eventually necessitates calcium release back into the cytosol[73]–[75]. Currently it is not known to what degree this mitochondrial calcium efflux may contribute to SR refilling or diastolic triggering of CICR, particularly if mitochondria have fused into networks[76]–[81].

Redox potential would appear to have a hermetic response curve suggesting various affinities for stimulatory and inhibitory regulatory pathways ultimately with ROS becoming toxic at high concentration. As the outcome of an intracellular calcium signal ordinarily depends on the strength, localisation, duration and pattern of that signal, SR Ca^2+^ release channeled to a tethered mitochondrion could act as a point source, propagating calcium signals via the mitochondrial network throughout the cell, obviating “local control”[57], [82]–[85]. Hence we postulate that Mfn-2 performs multiple, yet related, roles: tethering mitochondria to the SR, creating and maintaining calcium signalling at the MAM, modulating mitochondrial plasticity in response to local [Ca^2+^]_c_, transducing SIHM if stress can be ameliorated, and if not, initiating the death trigger[86]–[91]. This possibility raises the question “Could the MAM be a point source for arrhythmogenesis?”

### Limitations/Suggested further work

The C2C12 myotube model is derived from murine skeletal myoblasts and has the advantage of being capable of differentiation thus affords the opportunity to observe induced changes in mitochondrial plasticity while developing myofibrillar apparatus. This advantage of the C2C12 model readily permits reproducible interruption of ongoing fission/fusion processes while simultaneously offering the ability to investigate both fibrillatory and oxidative stress. In addition, the study of isolated myocytes permits the observation of autonomous intracellular processes, indicating that ATR can, at least in part, occur in the absence of cellular communication and does not require extracellular factors. However this inherent malleability is in direct contrast to that of terminally-differentiated human atrial myocytes. Unfortunately only relatively simple experiments can be designed examining scarce human atrial tissue from consenting patients.

We believe a more practical solution is to seek to rapidly identify potential candidate factors and regulatory pathways for investigation in the C2C12 myotube model, design and develop techniques for investigating these processes in differentiated atrial myocytes such as HL-1 myocytes and then perform simple corroborative experiments with human tissue.

## Conclusions

Long overlooked in cardiovascular pathology, the dynamic equilibrium of mitochondrial plasticity remains in equipoise under basal conditions. Cellular stress, whether as a result of rapid stimulation or from ROS promotes a hyperfused mitochondrial network, increased formation of mitochondria associated membrane and Mfn-2 mediated alterations in SR calcium handling with, as yet unknown, implications for arrhythmogenesis. Further work is necessary to determine the intermediate factors and ultimately the relevance of oxidative stress, mitochondrial plasticity and altered calcium handling in ATR.
